# Catalyzing communities of research rigour champions

**DOI:** 10.1093/braincomms/fcae120

**Published:** 2024-04-09

**Authors:** Audrey C Brumback, William X Q Ngiam, Dana M Lapato, David B Allison, Christin L Daniels, Michael Dougherty, Haley F Hazlett, Kara L Kerr, Susan Pusek, Melissa L Rethlefsen, Naomi Schrag, NINDS workshop Catalyzing Communities of Research Rigor Champions, Mathew Abrams, Eryn Adams, David B Allison, Juan Pablo Alperin, Gundula Bosch, Audrey Brumback, Damon Centola, Lique Coolen, April Clyburne-Sherin, Jennifer Croker, Sophia Crüwell, Christin Daniels, Michaela DeBolt, Ulrich Dirnagl, Michael Dougherty, Timothy Errington, Maryrose Franko, Anna Hatch, Kari Jordan, Kara Kerr, Halil Kilicoglu, Konrad Kording, Dana Lapato, Carole Lee, Daniella Lowenberg, Rebecca Lundwall, Malcolm MacLeod, Carmen Maldonaldo-Vlaar, Marcus Munafo, Alexandra Nelson, Nicole Nelson, William Ngiam, Sarah Nusser, Roger Peng, Jessica Polka, Russell Poldrack, Ishwar Puri, Susan Pusek, Pradeep Reedy Raamana, Pamela Reinagel, Melissa Rethlefsen, Jason Ritt, Joseph Ross, Karen Salt, Naomi Schrag, Thomas Steckler, Tracey Weissgerber, Alonzo Whyte, Jason Williams, Hao Ye

**Affiliations:** Department of Neurology, Dell Medical School at The University of Texas at Austin, TX 78712, USA; Department of Pediatrics, Dell Medical School at The University of Texas at Austin, TX 78712, USA; The Center for Learning and Memory, The University of Texas at Austin, TX 78712, USA; Institute of Mind and Biology, University of Chicago, IL 60637, USA; Department of Psychology, University of Chicago, IL 60637, USA; Department of Human and Molecular Genetics, Virginia Commonwealth University, Richmond, VA 23298, USA; School of Public Health, Indiana University Bloomington, IN 47405, USA; Triangle Center of Excellence in Regulatory Science and Innovation, University of North Carolina, Chapel Hill, and Duke University, Durham, NC 27599, USA; Department of Psychology, University of Maryland, MD 20742, USA; Declaration on Research Assessment (DORA), Rockville, MD 20852, USA; Department of Psychology, Oklahoma State University, OK 74078, USA; North Carolina Translational and Clinical Sciences Institute, University of North Carolina, Chapel Hill, NC 27599, USA; Health Sciences Library & Informatics Center, University of New Mexico, 87131, USA; Office of the Executive Vice President for Research, Columbia University, New York, NY 10027, USA

**Keywords:** open science, scientific rigour, reproducibility

## Abstract

The biomedical sciences must maintain and enhance a research culture that prioritizes rigour and transparency. The US National Institute of Neurological Disorders and Stroke convened a workshop entitled ‘Catalyzing Communities of Research Rigor Champions’ that brought together a diverse group of leaders in promoting research rigour and transparency (identified as ‘rigour champions’) to discuss strategies, barriers and resources for catalyzing technical, cultural and educational changes in the biomedical sciences. This article summarizes 2 days of panels and discussions and provides an overview of critical barriers to research rigour, perspectives behind reform initiatives and considerations for stakeholders across science. Additionally, we describe applications of network science to foster, maintain and expand cultural changes related to scientific rigour and opportunities to embed rigourous practices into didactic courses, training experiences and degree programme requirements. We hope this piece provides a primer for the wider research community on current discussions and actions and inspires individuals to build, join or expand collaborative networks within their own institutions that prioritize rigourous research practices.

## Introduction

According to the US National Institutes of Health (NIH), ‘scientific rigor is the strict application of the scientific method to ensure unbiased and well-controlled experimental design, methodology, analysis, interpretation, and reporting of results’.^1^ Rigour has always been a fundamental principle of credible and trustworthy science. Today, the application of rigour at every level of science has never been more important.^2^

How do we optimize rigour in scientific research? In May 2022, the National Institute of Neurological Disorders and Stroke (NINDS) convened a workshop entitled ‘Catalyzing Communities of Research Rigor Champions’ to bring together a diverse cross-sector of individuals identified as ‘rigour champions’—those who are actively pushing for reforms that promote rigour and transparency in biomedical research. This group included early career researchers, teaching and research faculty, department and institutional leaders and directors of non-profit community and governance organizations. Over 2 days of interactive panels and discussions, the group sought to identify critical strategies and approaches that will accelerate change in addressing what some have dubbed ‘the reproducibility crisis’ or the ‘replication crisis’ [though both terminologically and conceptually, the crisis label and narrative has been questioned (e.g. Wood and Wilson^3^)] through prioritizing research rigour and transparency (Box 1). This article summarizes those discussions, the perspectives of the rigour champions and current initiatives and resources promoting research rigour. Video recordings of the sessions are available on the NINDS website.

Box 1 **Workshop discussion topics**

**Behaviors**

What, operationally, do we want individuals and research groups to do?

What specific behaviors do researchers need to adopt to prioritize research rigor and transparency?

What evidence supports the need for these behaviors, and what knowledge, skills, and competencies do these behaviors require?

Which educational efforts are effective for developing and measuring these behaviors, knowledge, skills, and competencies?

**Working together**

What can we do as a group that we cannot do individually?

How can we know and use what other stakeholders (e.g., journals, funders, scholarly societies, professional organizations) are doing to help induce change?

What are the characteristics of ongoing movements and networks who seek to catalyze change as well as lessons learned in trying to organize groups?

How do we emulate these efforts, take advantage of developments in other sectors of our community, and build stronger alliances and networks?

**Motivation—developing partnerships and institutional buy-in**

When considering the changes that are needed to establish a culture of research rigor within institutions, which framing is effective (or ineffective) in eliciting support from allies and buy-in from colleagues and institutional leadership?

What are the links to positive aspirations and values that can inspire people to act?

**Facilitating change**

What can research groups and institutions do to make doing “the right thing” for research rigor and transparency the easiest, least burdensome path?

What are the types of tools that can make desired behaviors easier?

How do you remove counterincentives and barriers and develop incentives through norms and rewards that reinforce good behaviors?

There was broad agreement that efforts to improve science need to be diverse, collaborative and distributed across the entire research ecosystem. Reforms will need to span diverse organizations (e.g. research funding bodies, institutions, scientific journal publishers and scholarly societies) and individual career stages and expertise (e.g. early career researchers, junior and senior faculty and librarians). Reforms will need to improve all stages of the scientific process (i.e. research design, data collection/management/analysis and communication of findings). Efforts to improve science broadly fall into three categories: (i) technical (providing tools to aid rigourous and transparent research); (ii) cultural (changing incentives and values to prioritize research rigour); and (iii) educational (creating pedagogical resources for rigourous research training). We hope this article serves as an informative bulletin and encourages researchers to contribute to these reforms.

## Rigour: making scientific results trustworthy

To guide the conversation, Steven Goodman, leader of the Stanford Program on Research Rigor and Reproducibility, opened the meeting by providing a conceptual definition of rigour as ‘getting the uncertainty right’ in making claims about knowledge (i.e. reporting results). In a rigourous study, scientists assess and communicate the sources of uncertainty and bias and minimize them using best practices. What remains is a close fit between the methods and the certainty of the claim. A non-rigourous study would therefore be one in which the scientists claim more certainty than is deserved.

Walter Koroshetz, director of NINDS, further centred the conversation about rigour on the word ‘trustworthiness’. He stated that a major goal of optimizing scientific rigour is producing trustworthy results. The basis of science is to build on others’ work, so for science to move forward, scientists must be able to trust others’ results. Within small scientific communities, scientists think they know whose results are trustworthy and whose are sloppy. However, in our large global science network, it is not possible for scientists to know the trustworthiness of every author in the literature.

Dr. Koroshetz stated the current challenge: we must build trust in science. The penalties for not meeting this challenge are harsh. Lack of trustworthiness in the scientific literature sours the public’s enthusiasm and support for science, decreases the motivation for future generations to become scientists and discourages the pharmaceutical industry from developing treatments for even the most prevalent diseases like stroke. Policy change has its role, but building trust in science will require people in the trenches—champions of research rigour—to pick up the torch and change the system for the better.

An overarching theme of the discussions was how to define best practices for any given behaviour. Conference participants acknowledged that best practices are always evolving and are often discipline specific. Therefore, discussions centred on general principles and domains of behaviour, skills and competencies that transcend specific areas of research.

## The network science of widespread behaviour change

What is needed for champions of research rigour to influence others to transition from simply believing in rigour to practising daily routines of rigour (Box 2)? Damon Centola, PhD, professor of Communication, Sociology and Engineering at the University of Pennsylvania, provided a primer on the science of effecting widespread behavioural change. We frequently conceptualize spread through social networks using epidemiological thinking, which tells us that exposure to a contagion leads to its dissemination through the network. While this epidemiological/simple diffusion model may work well for some simple contagions like some viral diseases and viral videos, it does not work well for complex contagions such as shifting social norms. Because it involves shifting people out of typical known routines, mere exposure does not lead to transmission like it would with a simple contagion. In fact, Dr. Centola noted that nothing encounters more resistance than a change campaign that tries to shift us out of a behavioural routine we feel comfortable and confident in.

Box 2 **The Network Science of Behavior Change**

**There are no neutral interactions.** Non-adopters are countervailing influences by virtue of not adopting an improvement.

**It’s not about special people.** It’s about special places in the network where these people are and how those individuals can be sheltered from countervailing influences. And then having wide bridges between where an idea or practice is being tested and developed and other clusters to which that practice can spread.

**It’s not just about the PIs.** To establish and maintain communities connected by “wide bridges,” take advantage of the existing social networks at all levels of the research ecosystem (e.g., graduate students, postdoctoral scholars, research and lab staff, and principal investigators).

**Actively support change agents.** Cluster the hiring of change agents. Connect change agents to one another so they can reinforce each other as they influence the people in their local networks.

Therefore, in addition to exposure, positive social context and overcoming negative (or even neutral) peer feedback are required to drive the spread of complex behaviour change and social norms. Specifically, having several individuals act as bridges between communities seems to be most effective at spreading social norms from cluster to cluster. As an example, in the 1960s, Korea began a nationwide effort to encourage the use of contraception. Within the country, there were three types of communities: urban (one large cluster), clusters joined by ‘narrow bridges’ (one to two individuals linking clusters together) and clusters linked by ‘wide bridges’ (multiple individuals forming strong inter-cluster social connections). The most successful campaigns occurred in ‘wide bridge’ communities: villages that were socially connected through multiple linked individuals. Via a critical mass of social connections, when one cluster adopted the behavioural change, that new social norm spilled over to nearby clusters. By contrast, in the urban and ‘narrow bridge’ settings, the complex contagion of behavioural change never built the momentum needed for it to take hold and spread because it could not generate enough social support within or between clusters.

**Figure 1:**
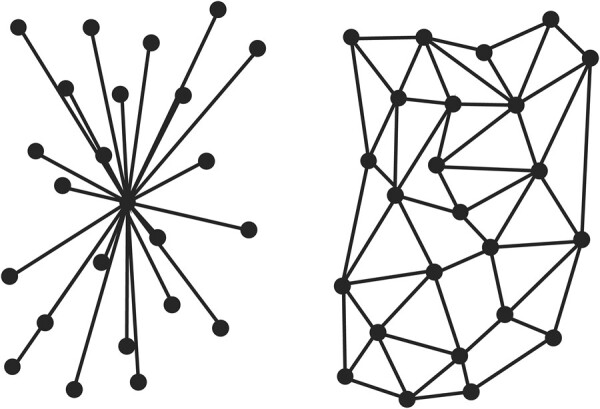
**Spread through social networks.** Left: exposure to a simple contagion (e.g. a virus) leads to dissemination throughout the network. Right: dissemination of complex contagions (e.g. shifting social norms, behavioural change) requires a self-reinforcing lattice of social connections.

Thus, to cause widespread shifts in social norms in science, building and maintaining multiple strong ties between scientific communities will likely be necessary to catalyze behavioural change on a large scale.^4^ Moreover, this social thinking model suggests that top–down reforms (e.g. changes in central policies) will need to be complemented by grassroots efforts of a network of scientists who identify as research rigour champions woven into communities (Fig. 1). This web of social reinforcements will allow the commitment to rigourous practices to spread between groups by reinforcing the legitimacy, credibility and relevance of the new social norms.

## Education and training

This section provides an overview of discussions on the approach to designing and evaluating research education/training efforts aimed at promoting rigourous research (Box 3). Discussants emphasized a need to instil foundational knowledge in practical statistics, study design principles and data management and offered examples from their home institutions. A common theme was that iterative improvement was valuable for achieving and maintaining incremental changes. However, the viewpoints and opinions expressed during these sessions were highly heterogeneous. While the importance of high-quality education, training and mentorship was undisputed, consensus was scarce regarding ‘how’ to achieve high-quality training experiences and even how to define it. People engaged in improving rigour need not work in isolation or create teaching materials from scratch. Existing educational resources and courses are listed in Table 1.

Box 3 **Education pearls of wisdom**

**Keep things positive.** Focus on the benefits of rigorous research rather than how being rigorous protects you from punishment.

**Encourage self-examination.** Ask how the scientist recognizes rigorous work within their area of specialization, then help the researcher identify strategies to produce similar rigor in their own work.

**Help with real-world implementation.** Offer ongoing support to help troubleshoot the application and dissemination of new knowledge and skills.

**It’s not one-and-done.** Incorporate training in scientific rigor in courses, workshops, and educational experiences.

**Learn from other systems.** Academic scientists could, for instance, benefit from the practices used by pharmaceutical companies or by champions of diversity, equity, and inclusion.

**The “good old days” weren’t that good.** By many indicators, science is more rigorous now, but we still have lots of room for improvement.

**Some rigor principles are agnostic to field; others aren’t.** Clearly advertise course content so learners can gauge whether the course will be appropriate for their needs.

**Make the content relevant.** Match the expertise of the instructors to that of the learners. Team teach if necessary. Provide practical examples that are congruent with the disciplines represented by students in the class.

**Embrace not having all the answers.** Encourage discussion.

**Appeal to values.** In the face of negative pressures, help scientists remember why they became scientists in the first place.

**Use natural incentives.** Help people understand how engaging in rigorous practices will save time and energy.

**Think beyond the tenure track.** Most PhD scientists are not on the tenure track. In designing incentives, think beyond the tenure track.

**Table 1:** Educational resources for promoting scientific rigour

Initiative	Link and description
Berlin Institute of Health (BIH) QUEST Center	https://s-quest.bihealth.org/fiddle/ This ‘match-making’ tool identifies alternate ways of publishing null or neutral results and data sets.
Reproducibility4Everyone	https://repro4everyone.org A community-led education initiative organizing introductory workshops on open research practices and collating resources that accelerate research.
Declaration on Research Assessment (DORA)	https://sfdora.org A set of recommendations to reform how the output of scientific research is evaluated. The general recommendation is not to use journal-based metrics, such as journal impact factors, as a proxy measure of research quality in the assessment of research articles or an individual researcher’s contributions for hiring, promotion or funding decisions.
Johns Hopkins R3 Center of Innovation in Science Education (R3ISE)	https://r3isenetwork.com A centre within Johns Hopkins Bloomberg School of Public Health providing graduate-level courses and programmes on rigour, responsibility and reproducibility. The research training courses include philosophy of science and research ethics.
The Carpentries	https://carpentries.org A non-profit education initiative providing workshops on computational and data science skills to researchers. Lessons are publicly available and actively maintained.
ASAPbio	https://asapbio.org A scientist-driven non-profit organization promoting the widespread use of preprints and transparent peer review.
UK Reproducibility Network	https://ukrn.org A national peer-led consortium coordinating the improvement of reproducibility and reliability of biomedical research in the UK. The model has since been adopted by researchers in many nations.
ReproducibiliTea	https://reproducibilitea.org A grassroots, early career researcher–led initiative to form open science journal clubs at academic institutions. The group provides resources to start communities, and the network now spans 23 countries.
Center for Open Science	https://cos.io An organization that takes a systems-level approach to increasing openness, integrity and reproducibility in research, such as by providing open infrastructure via the Open Science Framework for sharing data, code and preprints.
Higher Education Leadership Initiative for Open Scholarship (HELIOS)	https://heliosopen.org/ A cohort of US colleges and universities committed to advancing open scholarship.
Columbia University’s Research and Data Integrity (ReaDI) Program	https://research.columbia.edu/ReaDI-Program The Research and Data Integrity (ReaDI) Program is designed to enhance data management and research integrity at Columbia University. The ReaDI Program provides resources, outreach and consultation to researchers at all levels.
Duke Office of Scientific Integrity (DOSI) & Research Quality Management Program (RQMP)	https://research.duke.edu/dosi/; https://myresearchpath.duke.edu/topics/research-quality-management-program-rqmp The programme aims to strengthen research culture through approaches that are inclusive, comprehensive, multifaceted, pragmatic and empowering.
Community for Rigor	https://c4r.io The Community for Rigor funded by the NIH/NINDS to create an online educational resource to teach research rigour at a large scale.
Framework for Open and Reproducible Research Training	https://FORRT.org A community-powered initiative to form a nexus of pedagogical resources for rigourous research training. It provides a pedagogical infrastructure and didactic resource designed to recognize and support the teaching and mentoring of open and reproducible science.
Research Resource Identifiers Initiative	https://www.rrids.org Research Resource Identifiers (#RRID) are ID numbers assigned to help researchers cite key resources (antibodies, model organisms and software projects) in the biomedical literature to improve transparency of research methods.
Rigor resources compiled by NINDS	https://www.ninds.nih.gov/current-research/trans-agency-activities/rigor-transparency/rigor-champions-and-resources A table of hundreds of online rigour resources.
Initiative to Improve Education in the Principles of Rigorous Research	https://www.ninds.nih.gov/current-research/trans-agency-activities/rigor-transparency/initiative-improve-education-principles-rigorous-research NIH’s experimental science rigour initiative with funding to support education of principles of rigourous scientific research.
NINDS Office of Research Quality	https://www.ninds.nih.gov/current-research/trans-agency-activities/ninds-office-research-quality NINDS Office of Research Quality is dedicated to promoting experimental and analytical rigour, measures to reduce bias, transparent reporting and high-quality scientific research.
NINDS Rigorous Study Design and Transparent Reporting	https://www.ninds.nih.gov/funding/preparing-your-application/preparing-research-plan/rigorous-study-design-and-transparent-reporting A list of elements of rigourous and transparent experimental design that may be relevant to the NINDS research community and to emphasize NINDS’s interest in scientific rigour and transparency more broadly.
NINDS Sustainable Transformation of Institutional Research Rigor (STIRR) Initiative	https://www.ninds.nih.gov/current-research/trans-agency-activities/rigor-transparency/rigor-champions/ninds-sustainable-transformation-institutional-research-rigor-stirr-initiative This programme aims to support the establishment of programmes to enhance research rigour and transparency practices within academic and research institutions to promote a culture of high-quality neuroscience research.

One point of agreement was that revising coursework would not translate to behavioural change if the rigour practice trainees taught in the classroom are incongruent with the practices in their research environments. Thus, instilling behavioural changes in faculty and senior researchers (and the support to do so by the institution) was deemed critical to promote learning of rigourous research practices. It was suggested that rigourous research practices could be knitted into all layers of research training, such as coursework learning objectives, graduation requirements and awards for trainees and mentors. One idea was that trainees, as part their thesis proposal, would draft a protocol that—after committee feedback and revisions—would be submitted to protocols.io or uploaded as a preregistration to a platform like the Open Science Framework. Incorporating rigour checklists into dissertation committee meetings would then reinforce and promote rigourous practices throughout the lifecycle of a trainee’s project.

Another point of agreement was the central role of direct mentorship as part of research training. Most trainees receive one-on-one mentorship from their primary advisor. Within a training programme, this apprenticeship-style mentorship introduces variability in training quality. Suggested solutions ranged from requiring faculty to complete mentor and mentee training regularly, to increased monitoring and communication between trainees and department rigour liaisons, to expanded roles for dissertation or thesis committee members, and even to transitioning more concretely from having one primary mentor to having two or more primary mentors. The suggestion of shifting from a dyad mentorship model sparked significant interest; multiple primary mentors could mean more funding stability if faculty pooled resources to support trainees. Additionally, primary mentors from different fields or backgrounds could enrich training and mentorship while also giving students hands-on experience with team science. This excitement was tempered by the need to protect trainees from being overworked (e.g. expected to complete the equivalent of two dissertation projects) and from faculty disagreements about what work to prioritize.

Participants shared a variety of personal experiences and examples of successful and unsuccessful adjustments to training at their institutions. Many ideas required minimal resources and minimal to no changes in formal curriculum. Examples included enriching journal clubs, sharing current educational materials (e.g. syllabi, presentations, readings and assignments) and recognizing and awarding outstanding trainees, staff and faculty for engaging with rigourous and/or open science practices and tools. Journal club changes were especially popular as a way to discuss how rigourous best practices should be incorporated throughout the research life cycle. For example, journal clubs could serve as a forum to understand the benefits of interim research products like preregistration and preprints. As journal club attendees learn how to evaluate the rigour of published studies, they can implement rigour practices in their own work with the support of other journal club members.

Other suggested approaches required funding and protected time, such as organizing semi-annual workshops or retreats that emphasize rigour, career development and community building. Others shared experiences with hands-on training workshops (e.g. hackathons, practical guides for citation management and best practices for high-performance computing). Some suggested approaches required significant institutional support. Those who shared their journeys of revamping their curricula or degree programmes emphasized that large-scale structural changes are often best achieved in phases. Incremental and iterative implementation allows the opportunity for feedback, to evaluate the impact of changes and revise strategies. Examples of these approaches included changing the curriculum to enable microcredentialing, shifting degree programme evaluations like qualifying exams to competency-based assessments and refocusing didactic courses around central themes or infusing active learning techniques into courses traditionally taught as lectures.

The forum emphasized the importance of properly evaluating science education and training to establish rigourous research practices. Key discussions focused on the need for data on the prevalence and impact of current initiatives, measuring training impact and assessing trainees. Experts agreed that a successful research rigour course should lead to observable, measurable behaviours. This allows for direct evaluation of training impact. Additional evaluation methods include peer reviewing the training curriculum and conducting pre- and post-training surveys to gauge skill and understanding changes in trainees. The success of such interventions heavily depends on the institutional context, including resource allocation, value placed on the training and prioritization of rigour. There was also a focus on assessing research trainees. One proposal involved integrating a rubric of transparent and rigourous research practices into trainee evaluations. This approach aims to incentivize and track the adoption of rigourous research behaviours, setting an expectation for trainees to prioritize high-quality research.

Most clinical professionals participate in ‘continuing education’ activities because accruing continuing education credits is required for recertification of their clinical licences. Professional societies could similarly encourage researchers to obtain continuing education credits by bestowing special membership status to members who participate in continuing education activities. Earning continuing scientific education credits (CSEs) can incentivize participation in workshops, seminars and other events that teach or refresh rigour training.

## Considerations for different levels of organization

### Research groups

Research group leaders can spell out their rigourous research expectations and practices in a lab manual (e.g. statement on lab culture and expectations, https://github.com/alylab/labmanual/blob/master/aly-lab-manual.pdf). Group leaders can set and reiterate the expectations that not all results are expected to be ‘significant’ or ‘positive’. Modelling growth mindset and sharing ‘failures’ may nurture a positive culture that encourages transparency and healthy scepticism. To optimize scientific rigour and communication of ideas, group leaders can solicit reviews of work in progress, manuscripts and grant proposals from outside the research group prior to submission. Finally, collaborative review of lab notebooks, code and data organization can help all team members improve reproducibility and replicability (as currently defined by the National Academies^5^). To build a culture of trust, group leaders can encourage ‘supportive scepticism’ in group meetings by modelling a style of critique that encourages excellence and a growth mindset. Participants emphasized transparent recordkeeping, including detailed lab notebooks (electronic and/or hand-written) and standard operating procedure documents with versioning so they are living, evolving documents. We discussed that rigour in team science depends on sharing research resources such as reagents, code, data, protocols and standardized procedures for data format and management.

### Departments

At the department level, chairs play a unique role as agents of change. They can leverage their positions to guide policy development at the unit level, reinforce good behaviours through culture change and build incentive systems at all levels. They are uniquely positioned for bidirectional communication between higher-level administrators and their department community. Many chairs sit on councils with other chairs, communicate regularly with their deans and are responsible for writing promotion letters. These contexts provide levers of change. Specifically, department chairs can ensure that values around reproducibility, replicability, transparency and rigour are interwoven into job postings, included in annual reviews, embedded in promotion documents, included as part of internal funding initiatives and rewarded through departmental awards and recognitions.

### Institutions

Institutions can promote a culture that values rigour through several practical means. The recommendations ultimately focused on two main themes—first, aligning criteria to include research rigour for evaluation and assessment, and second, promoting an institutional culture that prioritizes research rigour.

Major US research institutions typically have research compliance and integrity officers who report to a senior research officer (SRO). The SRO is charged with ensuring the institution’s compliance with regulations and policies governing research and growing the institution’s research enterprise through strategic planning, recruiting and resource allocation. Promoting research rigour supports both of these missions. Funding agencies and other stakeholders should encourage promotion of rigour to be formally incorporated into these influential institutional roles.

SROs and research integrity and compliance officers can champion research rigour by setting the tone ‘from the top’ that the institution values rigour. SROs can support and develop institutional programmes that support rigour and incentivize researcher behaviours that promote rigour. The SRO can regularly communicate values through broadcast communications, websites and the publication of editorials promoting rigour, as well as during presentations to deans, department chairs and faculty.^6^ Ideally, in these communications, SROs would share specific examples of what it means to be a rigourous researcher rather than speak in general terms about rigour. Finally, SROs can influence hiring practices by advocating for detailed assessments of quality and rigour of faculty candidates’ work beyond number of publications and journal impact factor.^7^

With the support of the institution’s SRO, research integrity and compliance officers can implement initiatives to promote rigour, research quality, good data management and research integrity. The office’s programming could include development of tools and templates for researchers such as checklists for keeping a lab notebook, on-boarding and off-boarding resources; tutorials on data management; a repository of rigour and reproducibility literature; talks, trainings and journal clubs on data management; and research integrity for graduate students, postdocs, administrators and academic departments. In sum, SROs and research integrity and compliance professionals are key institutional champions for rigour. Many are likely taking steps to promote these values already.

### Consortia

Implementing reform in well-established systems of research culture is inherently challenging. The San Francisco Declaration for Research Assessment (DORA, https://sfdora.org) is one example of a mechanism to drive and maintain change at the institutional level. DORA is a global initiative that campaigns to improve research evaluation practices for hiring, promotion, tenure and funding decisions.^8^ The declaration provides recommendations to funding agencies, institutions, publishers, organizations supplying the metrics and researchers on how to implement more responsible research assessment practices that incentivize rigourous and transparent research. Journal-level metrics, such as journal impact factors, have often been used as proxy measures for a scientist’s research productivity and quality by academic institutions.^9^ DORA promotes a system of evaluating and rewarding work that promotes rigour, such as creating research protocols or providing accessible data sets. The use of narrative-style curricula vitae (CVs) can communicate how open science and rigour practices were incorporated into a researcher’s work.^10,11^

Case studies of successful reform can inspire action and provide practical strategies for change. DORA, in collaboration with SPARC Europe and the European University Association, created a case study repository for universities and national consortia. In many of the case studies, one common theme is the importance of communication among all stakeholders in building support for reform initiatives. However, the cases make clear that there is no ‘one-size-fits-all’ approach to reforming research culture. The case studies demonstrate several strategies for catalyzing buy-in, and DORA’s SPACE rubric provides a guide for rigour champions to tailor generalizable principles to their specific institutions.^12,13^

### Professional societies

Professional societies could set standards for transparency for conference posters and talks that report pilot or preliminary data.^14^ Society awards could focus on rigourous practices or include assessment of rigour in their criteria. Late-breaking abstract calls, which may promote rushed work and more mistakes, might be reconsidered. Society journals can promote taxonomy credit for contributor roles [e.g. through CRediT (Contributor Roles Taxonomy^15^)], publish failed replications, encourage registered reports (a type of publication designed to incentivize rigour and remove the publication bias towards novelty or positive results^16^), require the use of Research Resource Identifiers (https://www.rrids.org), encourage the use of estimation statistics, require authors to specifically describe methods used for masking the identity of experimental and control groups and require the use of multiple raters for subjective scoring measures. Societies could also provide support for new investigators on lab management skills, grantsmanship and collaborating with senior investigators to write grants.

### Publishers

Journals have tremendous power to immediately change practices through manuscript submission requirements. For instance, many journals now encourage the use of estimation statistics,^17^ data visualizations that show all data points^18,19^ and structured methods reporting.^20^ The advent of open-source data visualization and analysis programmes will decrease the barrier to implementing these approaches in research.

The Transparency and Openness Promotion (TOP) Guidelines were developed as a tool for journals to create policies aligned with best practices around open science and transparency.^21^ By allowing journals to pick their customized levels of support for each standard, the guidelines could help shift policies in a way that embeds them within their communities. Other tools at the journal level included support for registered reports^16^ and for Open Science Badges,^22^ which bring attention to and reward these research practices. Finally, helping authors find the right home for their publications can promote the dissemination of traditionally difficult-to-publish work such as null results or replication studies [see Berlin Institute of Health (BIH) QUEST Center below].

### Funders

If funding organizations required statements of specific methods for ensuring rigour and reproducibility in grant applications, it would set researchers up for success to use rigourous practices during the execution of the project. By requiring the use of rigourous practices from the inception of a project, researchers will be prepared to present data in a rigourous fashion at the time of publication.

Support for exploratory research, replication studies or for the curation and publication of protocols and data sets would incentivize these types of work. Finally, the generous use of no-cost extensions could ensure that projects reach their conclusions in a rigourous fashion rather than researchers rushing to produce a final product before funding ends.

## Areas of focus

### Hiring, promotion and tenure

Institutions and departments can prioritize rigour and open science in hiring of new faculty. Job ads can explicitly name rigour as a valued quality of successful applicants. Statements regarding research rigour can be required in job applications. Cohort hires of faculty who champion research rigour could quickly spread best research practices throughout a department.

The institutions could provide rigour training for all new faculty members. This can be used to communicate expectations and introduce available resources at the institution that promote rigour. The same small groups of new hires enrolled in the initial training could be maintained for subsequent trainings to create a sense of belonging, incentives and peer support.

There was broad agreement that including assessments of rigour in promotion and tenure evaluations would help incentivize the inclusion of rigour practices in academic research programmes. Promotion candidates could be required to include statements of the rigour practices they incorporated into their research. Internal and external reviewers could use a rubric provided by the university to assess rigour in their evaluations of candidates.

Regular evaluations of research practices, similar to peer teaching evaluations, could provide actionable feedback to researchers. Progress towards rigour goals could be discussed at annual and mid-tenure reviews. Encouraging the use of annotated CVs to document the rigourous practices used in individual publications could help provide a real-time record of a researcher’s commitment to open science and transparency.

Limiting the number of publications required for consideration of promotion or considering a researcher’s ‘n-best’ papers may help balance research output quality with quantity. To facilitate the inclusion of null or neutral results in CVs, departments can allow faculty to describe such studies, even if they were unable to get them published. To incentivize the use of and acknowledge the vital role non-publication-producing activities play in maintaining research quality, promotion and tenure policies can be adapted to include research credit for peer review activities as well as mentoring, training and teaching rigour.

### Other rewards

Promotion and tenure are only two of many incentives influencing researcher behaviour. Bonuses for disseminating work that complies with open science and rigour practices can motivate both trainees and principal investigators. These awards could recognize data reuse, preclinical research registration and publication of null results. Salary support could be provided for faculty members who perform internal reviews of manuscripts and grant applications or serve as rigour champions in other ways. Ensuring that even a small percentage of each faculty member’s full-time equivalent is dedicated to training in research rigour would communicate the institution’s commitment to upholding high standards of scientific integrity. Awards and fellowships to graduate students and postdoctoral scholars can help build and maintain a culture of rigour within training programmes. Departments can use department-level awards and friendly competition through inter-departmental bragging rights to encourage faculty and trainee use of rigour tools and practices.

### Environmental enrichment

Institutional facilities can be optimized to enhance rigour. For instance, animal care facilities could work with researchers to facilitate randomization and masking of experimental group identities. Encouraging use of institutional animal behaviour cores could facilitate comprehensive evaluations of animal behaviour and enhance reproducibility and replicability. Other institutional processes could be streamlined to minimize redundancy. For example, aligning written IRB and IACUC protocols with the formatting and content used in article methods sections could enable more detailed reporting of methods in publications.

Dedicated reproducibility staff can provide statistical and programming support to scientists and facilitate data pipeline management. Hiring PhD-level researchers into staff scientist positions instead of temporary postdoctoral positions could help decrease staff turnover and build continuity.

Social networks can help with the uptake and maintenance of new behaviours. Therefore, even simple interventions like providing food and beverages at activities promoting rigour may increase attendance and engagement.^23^

## Conclusion

The mission to make research rigour the norm in biomedical sciences requires individual and collective action. This article provides a broad overview on what rigour champions are currently thinking and doing to improve research practices in various aspects of science. We hope individuals explore the many recommendations and initiatives presented here and engage in the movement to strengthen science by prioritizing research rigour.

## Data Availability

Data sharing is not applicable to this article as no new data were created or analysed.

## Appendix

### Workshop organizers

Steven Goodman and Véronique Kiermer.

### Workshop participants

Mathew Abrams, Eryn Adams, David B. Allison, Juan Pablo Alperin, Gundula Bosch, Audrey Brumback, Damon Centola, Lique Coolen, April Clyburne-Sherin, Jennifer Croker, Sophia Crüwell, Christin Daniels, Michaela DeBolt, Ulrich Dirnagl, Michael Dougherty, Timothy Errington, Maryrose Franko, Anna Hatch, Kari Jordan, Kara Kerr, Halil Kilicoglu, Konrad Kording, Dana Lapato, Carole Lee, Daniella Lowenberg, Rebecca Lundwall, Malcolm MacLeod, Carmen Maldonaldo-Vlaar, Marcus Munafo, Alexandra Nelson, Nicole Nelson, William Ngiam, Sarah Nusser, Roger Peng, Jessica Polka, Russell Poldrack, Ishwar Puri, Susan Pusek, Pradeep Reedy Raamana, Pamela Reinagel, Melissa Rethlefsen, Jason Ritt, Joseph Ross, Karen Salt, Naomi Schrag, Thomas Steckler, Tracey Weissgerber, Alonzo Whyte, Jason Williams and Hao Ye.
