# Correction to: Catalyzing communities of research rigour champions

**DOI:** 10.1093/braincomms/fcae242

**Published:** 2024-07-24

This is a correction to: Audrey C Brumback, William X Q Ngiam, Dana M Lapato, David B Allison, Christin L Daniels, Michael Dougherty, Haley F Hazlett, Kara L Kerr, Susan Pusek, Melissa L Rethlefsen, Naomi Schrag, NINDS workshop Catalyzing Communities of Research Rigor Champions, Catalyzing communities of research rigour champions, *Brain Communications*, Volume 6, Issue 3, 2024, fcae120, **https://doi.org/10.1093/braincomms/fcae120**

In the originally published version of this paper the author Melissa L Rethlefsen was inadvertently omitted. This error has been corrected and the Acknowledgements section updated to reflect Melissa L Rethlefsen's contributions.

